# Crocin exerts anti-inflammatory and anti-arthritic effects on type II collagen-induced arthritis in rats

**DOI:** 10.1080/13880209.2018.1448874

**Published:** 2018-03-15

**Authors:** Wei Liu, Yufeng Sun, Zhenping Cheng, Yong Guo, Peiming Liu, Ying Wen

**Affiliations:** Department of Orthopaedics, The Fifth Hospital of Harbin, Harbin, Heilongjiang, China

**Keywords:** Rheumatoid arthritis, autoimmune disease, joint destruction

## Abstract

**Context:** Rheumatoid arthritis (RA) is a common systemic auto-immune disease, which is characterized by chronic and symmetry synovial inflammation. Crocin has been reported to exhibit anti-inflammatory effects in animal models.

**Objective:** This study investigates the anti-inflammatory and anti-arthritic effects of crocin on type II collagen-induced arthritis (CIA) in Wistar rats.

**Materials and methods:** The CIA rat model was established and randomly divided into five groups with or without crocin treatment (10, 20 or 40 mg/kg), which was started on day 21 after arthritis induction and persisted for 36 days. The symptoms and molecular mechanisms of CIA and crocin-treated CIA rats were compared and investigated.

**Results:** CIA rats presented severe RA symptoms, including high arthritis score, paw swelling, joint inflammation, bone erosion, chondrocyte death, cartilage destruction, enhanced expressions of matrix metalloproteinase (MMP) and pro-inflammatory cytokines. However, crocin could mitigate these symptoms. Crocin (40 mg/kg) exhibited the most efficient therapeutic function on CIA rats: the histological scores of joint inflammation, bone erosion, chondrocyte death, cartilage surface erosion, and bone erosion of CIA rats receiving 40 mg/kg crocin treatment were comparable to the normal rats. MMP-1, -3 and -13 protein expression levels of CIA rats with 40 mg/kg crocin treatment were decreased to levels similar to normal rats. Moreover, crocin could also inhibit the expression of TNF-α, IL-17, IL-6 and CXCL8 in serum and ankle tissues of CIA rats.

**Conclusions:** In summary, crocin exhibits therapeutic potential for RA, by mitigating the symptoms and inhibiting the pro-inflammatory factor expression.

## Introduction

Rheumatoid arthritis (RA) is a common systemic auto-immune disease characterized by chronic and symmetry synovial inflammation, which potentially leads to irreversible joint destruction (Nakken et al. 2017). Most auto-immune diseases result from targeting of autologous proteins by auto-reactive T cells and antibodies in both human and animal models (Cho et al. 2007). Type II collagen (CII) is one of the major auto-antigens in human RA, as the incidences of anti-CII antibodies and CII-specific T cells are relatively high (Kim et al. 1999, 2000). The immunity generated against CII of heterologous species usually results in joint destruction, since CII is exclusively expressed in the joints articular cartilage (Wooley and Chapedelaine 1987; Durie et al. 1994). Type II collagen-induced arthritis (CIA) animal models have been widely used to study human RA, as they demonstrate that auto-immunity to CII can lead to auto-immune arthritis, with symptoms such as bone erosion, cartilage destruction and synovial joints (Cho et al. 2007).

T cells consist of 75–90% of the lymphocytic infiltrate in the inflamed synovial membrane of RA patients (Bankhurst et al. 2008). As RA has been reported to be associated with major histocompatibility complex (MHC) class II genes (Griffiths et al. 1992; Rosloniec et al. 1996), it is speculated that T cells play an essential role in the pathogenesis of RA, which is supported by experimental evidence: mice with CD4 deficiency were less susceptible to CIA compared to wild-type mice (Ehinger et al. 2001). Recently, Th17 cells were considered as the major players of CIA pathogenesis (Murphy et al. 2003). Th17 cells, producing cytokines such as interleukin (IL)-17 and IL-22, could be stimulated by IL-23, IL-6 and transforming growth factor β (TGF-β), and their differentiation is inhibited by interferon γ (IFN-γ) (Zhu et al. 2010). IL-17 initiates inflammation by increasing the generation of pro-inflammatory cytokines, such as tumour necrosis factor alpha (TNF-α), IL-1β and IL-6 (Peck and Mellins 2009), and IL-17 maintains detectable levels in RA synovium (Chabaud et al. 1999). Studies also support the essential role of Th17 in CIA, where antibody neutralizing the endogenous IL-17 or IL-22 deletion could alleviate RA symptoms (Lubberts et al. 2004; Geboes et al. 2009; Kelchtermans et al. 2009).

The synovial macrophages are also associated with articular destruction in joints of RA (Mulherin et al. 1996). Together with other attracted monocytes, the synovial macrophages are responsible for angiogenesis, matrix degradation and cytokine production. TNF-α and IL-6 are among the most important cytokines produced by synovial macrophages (Schurgers et al. 2011). The fibroblast-like synoviocytes (FLSs), one of the main structures that constitute the intimal lining layer of the synovium, are the major degraders of cartilage. FLSs release cathepsins, aggrecanases and matrix metalloproteinase (MMP) to degrade the cartilage after stimulated by cytokines such as TNF-α and IL-6 (Scott et al. 2010).

Thus, overcoming the inflammation initiated by CII-induced immunity is critical for RA therapy. Recently, numerous studies have focused on the therapeutic roles of natural products, by isolating and investigating the bioactive components from the natural sources such as vegetables, fruits and herbs. *Crocus sativus* L. (Iridaceae) has attracted a great deal of research attention for its numerous beneficial properties, of which crocin is one of the most powerful components (Bhandari 2015). Previous studies revealed that crocin had therapeutic potential in several diseases and physiological disorders including Alzheimer’s disease, digestive disorders, nervous system disorders and even cancer chemoprevention (Bhandari 2015; Khazdair et al. 2015; Khorasany and Hosseinzadeh 2016; Finley and Gao 2017). Furthermore, crocin has been reported to exhibit anti-inflammatory effects in animal models (Li et al. 2015, 2017; Samarghandian et al. 2016).

In our study, we established the CIA rat model, and treated the CIA rats with different doses of crocin to investigate the anti-inflammation and anti-RA effects of crocin.

## Materials and methods

### CIA animal model

Lyophilized bovine CII (Invitrogen, Carlsbad, CA) was emulsified in complete Freund’s adjuvant (CFA) (Merck KGaA, Darmstadt, Germany) with the ratio of 1:1 for the booster dose, and in incomplete Freund’s adjuvant (IFA) (Merck KGaA, Darmstadt, Germany) with the ratio of 1:1 for booster dose, and the final concentration was 2 mg/mL. A volume of 0.2 mL suspension, including 0.2 mg collagen, was injected subcutaneously at four sites of the tail base. On day 6 after the first injection, 0.1 mL of booster dose suspension was injected avoiding the original site. CIA rats were kept for 21 days for observing the symptoms of arthritis. Oral treatment with the crocin was started on day 21 after arthritis induction and persisted for 36 days. The protocols were approved by the Committee of Animal Care at the Fifth Hospital of Harbin.

### Drug administration strategy

All the experimental rats were randomly divided into five groups, with each group consisting of five rats. Group I: saline control, receiving saline treatment; Group II: CIA control, suffering CIA and received no treatment; Group III: CIA rats treated with 10 mg/kg crocin; Group IV: CIA rats treated with 20 mg/kg crocin, and Group V: CIA rats treated with 40 mg/kg crocin.

### Histology

Rats were sacrificed at day 36 after the CII introduction. The ankle joints were harvested, fixed in 10% formalin for four days, decalcified with 5% formic acid, embedded in paraffin and sectioned for 7 μm. Haematoxylin and eosin (H&E) staining was performed to investigate the joint pathology. Histologic severity was scored using four different parameters: joint inflammation, chondrocyte death, cartilage surface erosion and bone erosion.

### Western blotting

The serum levels of MMP-1, MMP-3 and MMP-13 were estimated by western blot analysis using a standard protocol. Briefly, the protein concentration of the serum samples was quantified by BCATM Protein Assay Kit (Pierce, WI). Then, the serum samples were separated by sodium dodecyl sulphate-polyacrylamide gel electrophoresis (SDS-PAGE) and transferred to a polyvinylidene fluoride (PVDF) (Millipore, Billerica, MA) membrane. Following incubation 1 h with 5% nonfat milk at room temperature, the membranes were then incubated in the specific primary antibodies against MMP-1, MMP-3 and MMP-13 (Santa Cruz Biotechnology, Dallas, TX) overnight at 4 °C. The membranes were then incubated in the secondary antibodies (Santa Cruz Biotechnology, Dallas, TX) for 1 h at room temperature. Positive signal was developed by electrochemiluminescence (ECL) (Pierce, WI). The intensity of bands was determined by densitogram and β-actin was used as loading control.

### Determination of serum cytokine levels

Serum samples were incubated under ice-cold conditions and then centrifuged at 1800×*g* for 15 min. Next, the serum levels of TNF-α, IL-17, IL-6 and CXCL8 were determined by using the commercial enzyme-linked immunosorbent assay (ELISA) kits (Invitrogen, Carlsbad, CA) according to the manufacturer’s protocols and instructions.

### RNA extraction and cDNA synthesis

Total RNA from ankle tissue homogenate from each rat was isolated using TRIzol reagent (Invitrogen, Carlsbad, CA) according to the manufacturer’s instructions. cDNA was synthesized by using the cDNA Reverse Transcription Kit (Applied Biosystems, Waltham, MA) according to the manufacturer's instructions.

### Quantification of mRNA expression in ankle tissue by quantitative polymerase chain reaction (qPCR)

Quantitative analysis of the target genes expression was performed by qPCR by subjecting the resulting cDNA from the above preparation. The qPCR data were analysed using the relative gene expression method, represented by ^ΔΔ^CT as described in User Bulletin No. 2 (Applied Biosystems, Waltham, MA). The data were presented as the fold change in gene expression normalized to the endogenous reference gene (GAPDH) and relative to a calibrator.

### Statistical analysis

Results were represented as the mean ± SE with at least three experiments. Data were compared by one-way ANOVA followed by the Tukey–Kramer post-test and Student’s *t*-test, as appropriate. A *p* value of <0.05 was considered to be of statistical significance.

## Results

### Crocin decreases arthritis scores, paw swelling and weight loss in CIA rats

**Figure 1.**
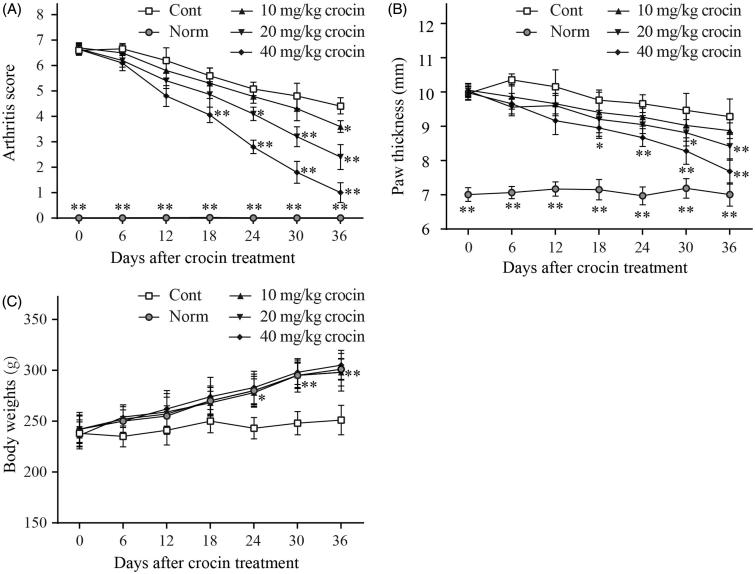
The effects of crocin treatment (10, 20 and 40 mg/kg/days) on arthritis score (A), paw swelling (B) and weight loss (C) in collagen-induced arthritis (CIA) rats. The arthritis score (A) and body weight (C) in each treatment group was determined daily. Paw swelling (B) was measured daily using microcallipers, and the mean width of the first hind paw in which arthritis developed in each treatment group was determined. Day 0 in the figure is day 28 after initial immunization. Norm: normal rats; Cont: control CIA rats. Data are presented as the mean ± SE (*n* = 15). *p < 0.05, **p < 0.01, compared with the Cont.

The CIA rat model was established to investigate the therapeutic impact of crocin on RA. When immunized with the CII, the CIA rats showed significant RA symptoms compared with normal rats, such as high arthritis scores, paw swelling and weight loss. Hereby, we treated the CIA rats with different doses of crocin (ranging from 10 to 40 mg/kg) for 36 days. Our results indicated that after the initial crocin administration, arthritis scores decreased in CIA rats compared to the control group (Figure 1(A)). In addition, after crocin treatment, the paw swelling of CIA rats was also relieved compared to the control group (Figure 1(B)). Notably, crocin influenced the arthritis scores and paw swelling in a dose-dependent manner. During the crocin treatment period, body weight of the control rats remained the same, while that of the normal rats underwent a normal increase. When the CIA rats were treated by crocin, their body weight exhibited a similar increasing trend as the normal rats (Figure 1(C)).

### Crocin decreases inflammation, chondrocyte death, cartilage surface erosion and bone erosion in CIA rats

**Figure 2.**
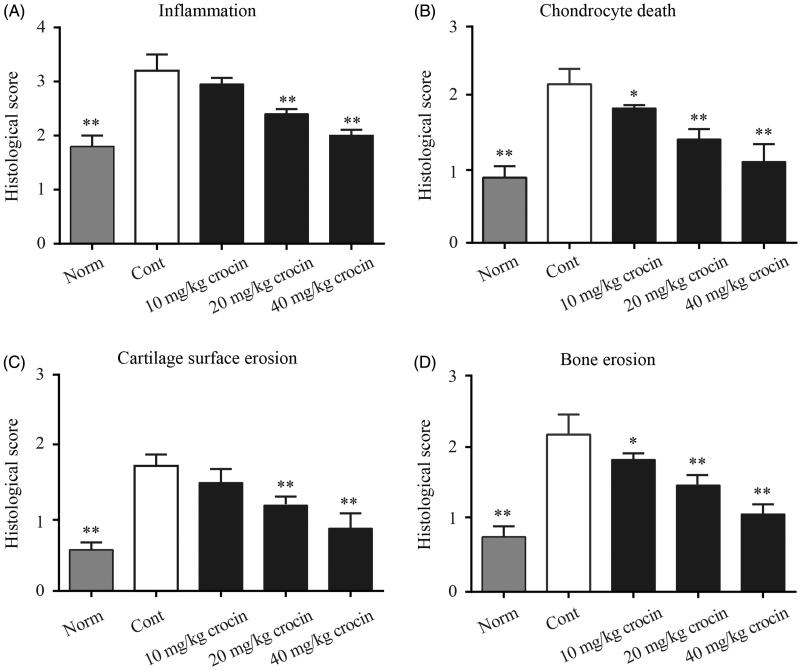
The effects of crocin treatment on joint inflammation, bone erosion and cartilage destruction in CIA rats. The ankle joints were subjected to histological analysis and inflammation (A), chondrocyte death (B), cartilage surface erosion (C), bone erosion (D) on day 36 after crocin treatment. Norm: normal rats; Cont: control CIA rats. Data are presented as the mean ± SE (*n* = 15). *p < 0.05, **p < 0.01, compared with the Cont.

Next, we utilized H&E staining to observe the synovial tissue hyperplasia conditions in the joint cavity. Rats of the CIA group underwent obvious synovial tissue hyperplasia, with vast infiltration of pro-inflammatory immune cells. While rats in normal group showed clear structure of joint synovial tissue with no hyperplasia, rats treated with 10 mg/kg crocin exhibited plenty of pro-inflammatory immune cell infiltration with synovial tissue hyperplasia. Crocin (20 mg/kg) administration reduced pro-inflammatory immune cell infiltration in the joint cavity and inhibited synovial tissue hyperplasia. When the doses of crocin were increased to 40 mg/kg, almost no pro-inflammatory immune cells were observed in the joint cavity, and synovial tissue hyperplasia were also significantly reduced (Supplementary Figure S1). The statistical data of the inflammation histological scores are shown in Figure 2(A), which indicated that crocin exhibited inhibitory effects on CIA articular inflammatory lesions. Furthermore, we found that crocin could also decrease chondrocyte death, cartilage surface erosion and bone erosion in CIA rats in a dose-dependent manner (Figure 2(B–D)).

### Crocin inhibits serum levels of MMP-1, MMP-3 and MMP-13 in CIA rats

**Figure 3.**
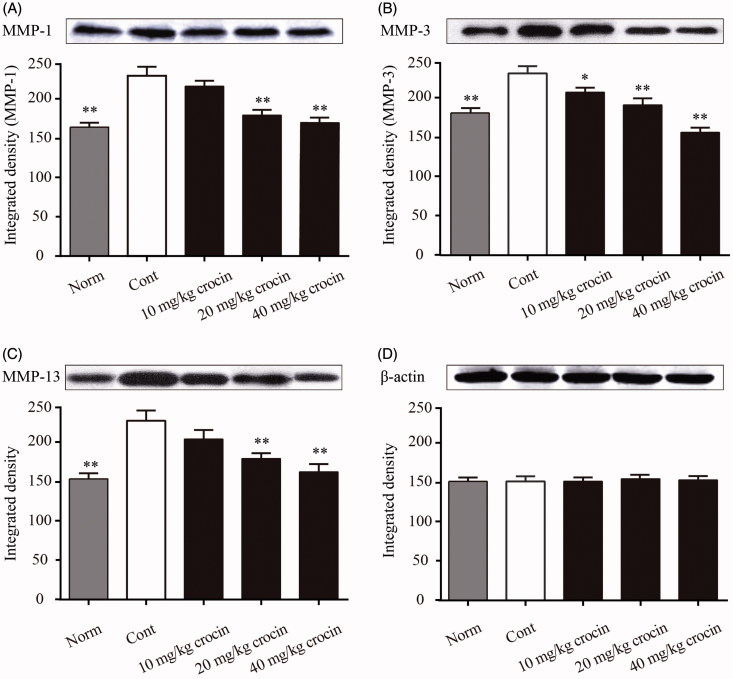
The effects of crocin on protein expression of MMP-1, MMP-3 and MMP-13 in serum in CIA rats. Serum samples were separated by SDS-PAGE and MMPs levels were measured by immunoblotting. The figure represents the protein expression levels and the corresponding densitogram of MMP-1 (A); MMP-3 (B); MMP-13 (C) and β-actin (D). Norm: normal rats; Cont: control CIA rats. Data are presented as the mean ± SE (*n* = 15). *p < 0.05, **p < 0.01, compared with the Cont.

The MMP family proteins are associated with the breakdown of extracellular matrix proteins during multiple physiological processes including embryonic development, tissue remodelling or disease processes such as arthritis and tumour metastasis. Therefore, we analysed the serum levels of MMP-1, MMP-3 and MMP-13 in CIA rats. The Western blot results showed that the serum levels of MMP-1, MMP-3 and MMP-13 in the CIA rats were dramatically elevated compared with those of the normal rats (Figure 3(A–D)). However, we found that daily treatment of CIA rats with crocin significantly decreased the serum levels of MMP-1, MMP-3 and MMP-13. When the dose of crocin reached the highest level (40 mg/kg), it exerted the most prominent inhibitory effect on MMP-1, MMP-3 and MMP-13 expression (Figure 3(A–D)).

### Crocin decreases the serum levels of TNF-α, IL-17, IL-6 and CXCL8 in CIA rats

**Figure 4.**
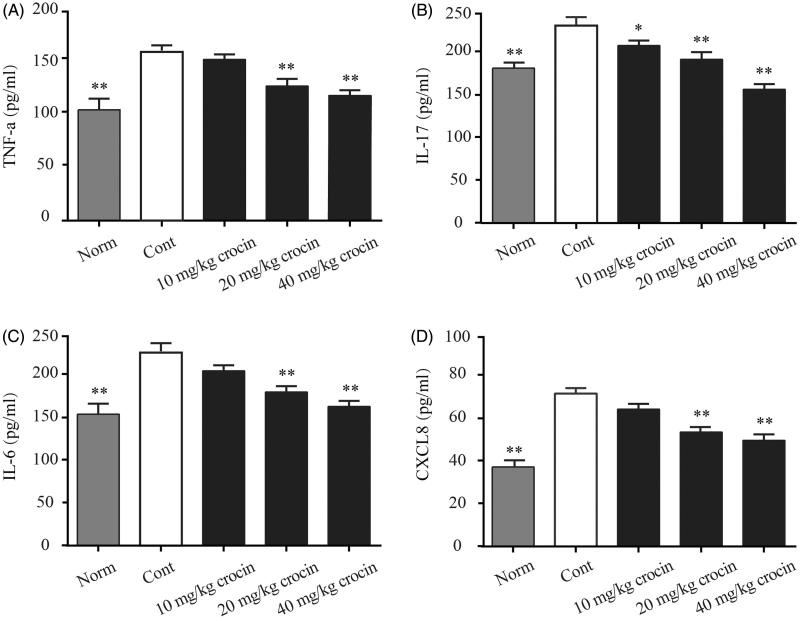
The effects of crocin on the release of TNF-α, IL-17, IL-6 and CXCL8 into the serum of CIA rats. (A) TNF-α content; (B) IL-17 content; (C) IL-6 content; (D) CXCL8 content. Norm: normal rats; Cont: control CIA rats. Data are presented as the mean ± SE (*n* = 15). *p < 0.05, **p < 0.01, compared with the Cont.

As we have found that crocin could relieve the CIA-induced inflammation, we then investigated the inflammatory cytokine levels in the serum of CIA rats. Here, we determined the serum levels of TNF-α, IL-17, IL-6 and CXCL8 by ELISA, and found that the CIA rats showed obviously enhanced levels of these cytokines, compared with rats from the normal group (Figure 4(A–D)). Consistent with our previous findings, we found that when the CIA rats received crocin treatment at different doses, serum levels of these pro-inflammatory cytokines decreased in different extents. The CIA rats received the highest dose (40 mg/kg) of crocin exhibited lowest serum levels of pro-inflammatory cytokines, compared with other experimental groups (Figure 4(A–D)).

### Crocin downregulates mRNA expression ofpro-inflammatory cytokines

**Figure 5.**
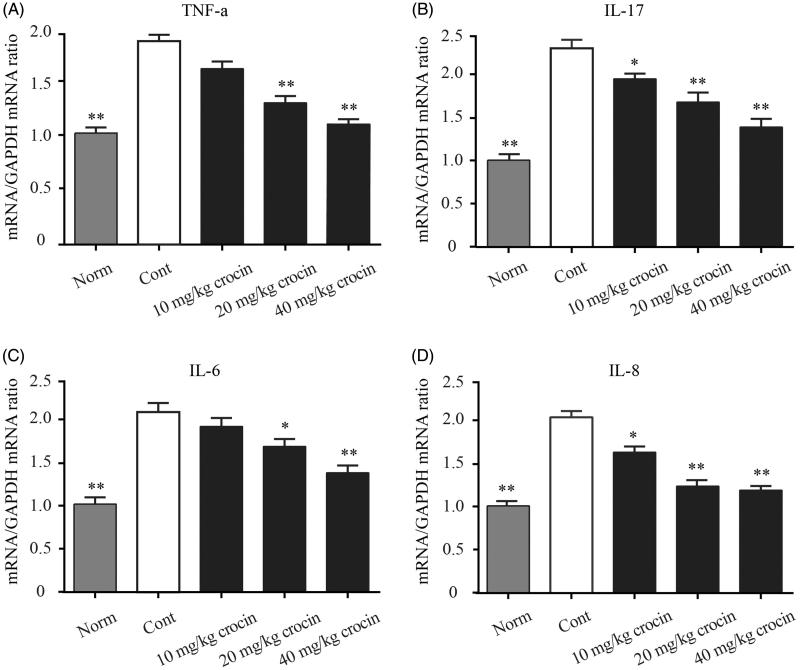
The effect of crocin on the gene expression of pro-inflammatory cytokines. mRNA expression was measured by quantitative RT-PCR in the ankle tissue in CIA rats. (A) TNF-α content; (B) IL-17 content; (C) IL-6 content; (D) CXCL8 content. Statistical analysis was performed using one-way ANOVA followed by the Tukey–Kramer post-test. Norm: normal rats; Cont: control CIA rats. Data are presented as the mean ± SE (*n* = 15). *p < 0.05, **p < 0.01, compared with the Cont.

Finally, we explored the mRNA expression levels of the above-mentioned pro-inflammatory cytokines (TNF-α, IL-17, IL-6 and CXCL8) in the ankle tissues of CIA rats. The control group showed significantly higher mRNA levels of these pro-inflammatory cytokines in the ankle joint, compared with that of the normal group (Figure 5(A–D)). On the other hand, crocin resulted in decreased mRNA expression levels of pro-inflammatory cytokines in the ankle tissues of CIA rats, in a dose-dependent manner (Figure 5(A–D)).

## Discussion

Both genetic and environmental factors contribute to the pathogenesis of RA, but to date there has been no cure for this auto-immune disease. Over 100 RA-associated loci, including the MHC II region, have been identified by genome-wide association studies. However, these loci present only a fraction of the genetic variance of RA, and it is still obscure how these loci function in the development and progression of RA. Among various environmental risk factors, smoking contributes significantly to RA development (Yau and Holmdahl 2016). Animal models are widely used to study the mechanisms of human RA, as they could properly mimic the polygenic nature and environmental-factor-dependence of this disease. CIA is one of the most commonly used models of RA, in which various cartilage-derived proteins, such as CII and type XI collagen (CXI) could be used to elicit arthritis in animals (Trentham et al. 1977; Lu et al. 2002).

In our study, we established the CIA rat model, and the rats induced by CII manifested severe RA symptoms, including high arthritis score, paw swelling (Figure 1(A,B)), joint inflammation, bone erosion, chondrocyte death and cartilage destruction (Figure 2(A,D)). Twenty-one days after CIA induction, we treated the CIA rats with three different doses of crocin. Crocin is an active water-soluble monomer component separated from the Chinese traditional medicine saffron. Several studies have reported that crocin possesses various therapeutic effects, such as antitumor effects, anti-atherosclerosis and antilipid peroxidation. Furthermore, crocin has been reported to have anti-inflammatory effect, and can be used as adjunctive therapy in many inflammatory diseases (Li et al. 2017). However, there are few studies focusing on the effects of crocin on RA.

In 2017, Li et al. found that crocin administration dramatically mitigated paw swelling of RA rats, reduced arthritis score in crocin treatment groups compared to that of the RA control group. Moreover, crocin treatment also remarkably decreased the serum levels of TNF-α, IL-1β and IL-6 in RA rats (Li et al. 2017). Hemshekhar et al. (2012) found that crocin suppressed the enhanced expression of bone joint exoglycosidases, tartrate resistant acid phosphatases and cathepsin-D, hence protecting against bone erosion of arthritic rats. In addition, they reported that crocin efficiently neutralized the elevated serum levels of inflammatory mediators, including enzymatic (MMP-3, MMP-13 and MMP-9 and HAases) and non-enzymatic factors (COX-2, IL-6, NF-κB, IL-1β, TNF-α and ROS) (Hemshekhar et al. 2012). These studies indicated that crocin might be an efficient anti-arthritis agent. In our present study, we found that crocin treatment significantly ameliorated the arthritis symptoms in CIA rats, including decreasing arthritis score, alleviating paw swelling (Figure 1(A,B)), mitigating joint inflammation, bone erosion, chondrocyte death and cartilage destruction (Figure 2(A,D)), in a dose-dependent manner. Subsequently, we further explored the molecular mechanisms underlying the anti-inflammatory effects of crocin.

MMPs are a family of enzymes responsible for the degradation of extracellular matrix molecules, which are efficient regulators of physiological tissue remodelling, and have been implicated in several diseases including RA. Based on their mediator functions, MMPs are desirable biomarkers in the diagnosis and evaluation of these diseases (Rose and Kooyman 2016). There are seven MMPs reported to be expressed in the articular cartilage, including MMP-1, MMP-2, MMP-3, MMP-8, MMP-9, MMP-13 and MMP-14, among which MMP-1, MMP-2, MMP-13 and MMP-14 are constitutively expressed in adult cartilage and are correlated with tissue turnover. While the cartilage MMP-3, MMP-8 and MMP-9 are expressed only in pathologic circumstances (Chubinskaya et al. 1999).

MMP-1 degrades the collagen types I, II and III, the expression levels of which are low under healthy circumstances. On the other hand, under arthritic conditions, MMP-1 expression is significantly upregulated and plays an important role in collagen degradation (Wu et al. 2008). MMP-3 has the capability to degrade collagen types II, III, IV, IX and X, as well as fibronectin, laminin, elastin and various proteoglycans. It is involved in wound healing, and is typically expressed in fibroblasts and epithelial cells in response to inflammatory compounds. As MMP-3 is normally absent in normal joint tissues, it could serve as a biomarker for arthritis (Kubota et al. 1997). MMP-13 is the most studied MMP member, as it exhibits robust capability to degrade CII that predominates in the articular cartilage. Even though MMP-13 principally targets CII, it could also degrade other types of collagens and matrix molecules such as proteoglycan, proteoglycan and perlecan (Shiomi et al. 2010). Therefore, we investigated the serum expression levels of representative MMP molecules (MMP-1, MMP-3 and MMP-13) in CIA rats. Western blot results showed that the serum contents of all the three MMPs were significantly increased in CIA rats, compared to the normal rats. When the CIA rats received crocin treatment, they showed decreased levels of MMP-1, MMP-3 and MMP-13 in a dose-dependent manner (Figure 3(A–C)). These results are consistent with previous findings.

Under RA conditions, immune cells such as macrophages, fibroblasts, dendritic cells, B cells, T cells and granulocytes constitute the inflamed synovial tissue, which destroy cartilage and bones. The synovial tissue inflammation results in increased angiogenesis facilitating the recruitment of inflammatory cells. These invading cells are the major source of pro-inflammatory cytokines, such as TNF-α, IL-6 CXCL8 and IL-17. These pro-inflammatory cytokines could in turn promote inflammation in the affected joint (Herman et al. 2008). Based on these previous findings, we detected levels of these pro-inflammatory cytokines (TNF-α, IL-6, CXCL8 and IL-17) in both serum and ankle tissue in our CIA rat model. We found that the expression of pro-inflammatory cytokines indeed increased in both serum and ankle tissue in CIA rats, compared to the normal rats. However, when the CIA rats received crocin administration, the pro-inflammatory cytokine levels decreased in a dose-dependent manner, and when the dose of crocin reached 40 mg/kg, these cytokine levels were almost reduced to the normal level (Figures 4 and 5).

In conclusion, we established the CIA rat model to investigate the therapeutic effect of crocin, the main constituent of saffron, on RA. Our CIA rat model manifested severe RA symptoms, including high arthritis score, paw swelling, joint inflammation, bone erosion, chondrocyte death, cartilage destruction, as well as enhanced MMPs and pro-inflammatory cytokines. However, crocin treatment could markedly mitigate all of the above symptoms and inhibit the expression of MMPs and pro-inflammatory cytokines. Our study indicated that crocin might have potent therapeutic potential for RA.

## Supplementary Material

Supplementary Figure S1
