# Supplementary material for: Crocin exerts anti-inflammatory and anti-arthritic effects on type II collagen-induced arthritis in rats
Source: Pharm Biol. 2018 Mar 15;56(1):209–16. doi: 10.1080/13880209.2018.1448874 (PMC6168764; doi:10.1080/13880209.2018.1448874)
Supplement: Supplementary Figure S1 [file IPHB_A_1448874_SM9303.docx]

**Supplementary Materials**


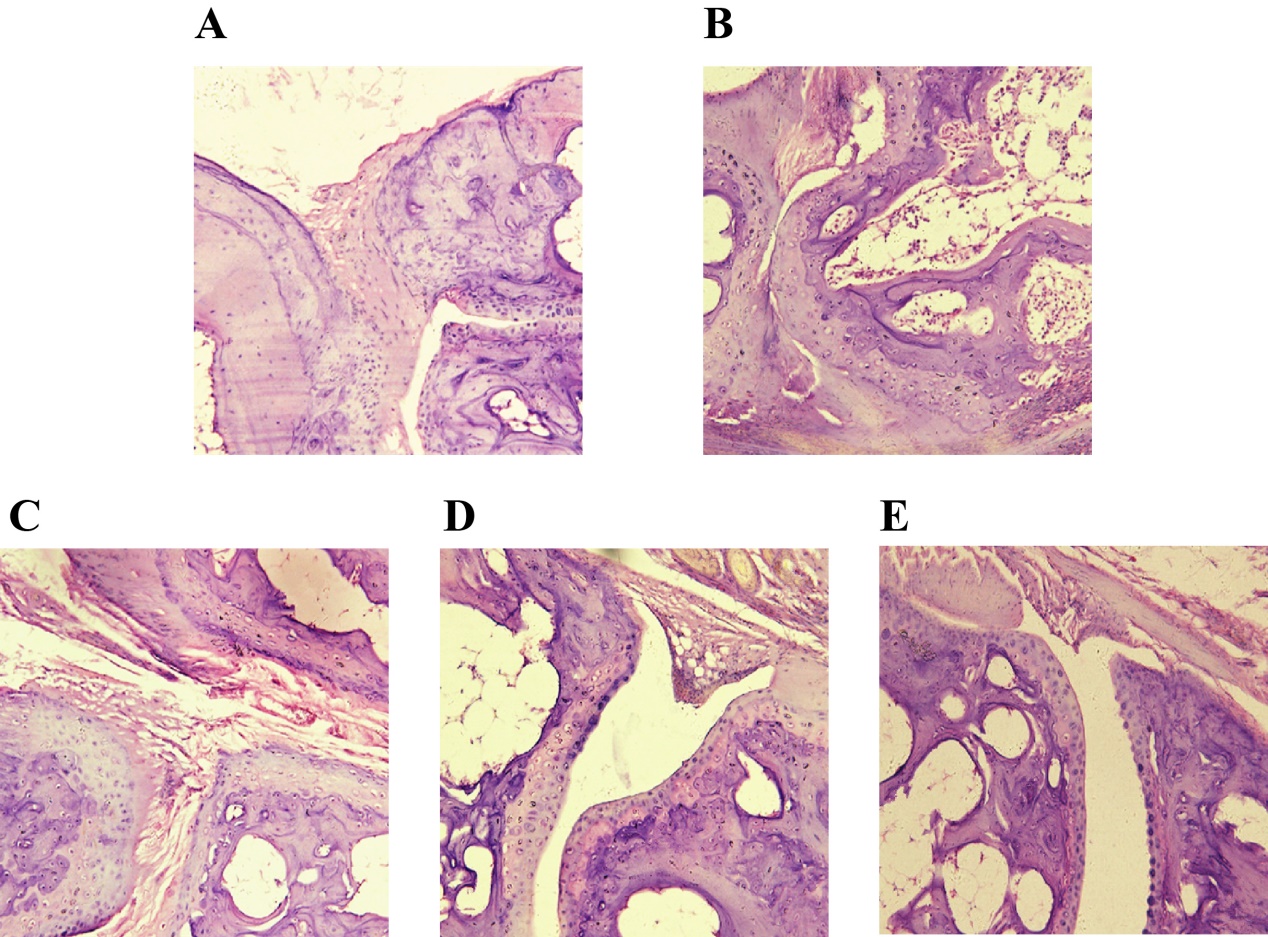


**Supplementary Figure S1**. The effects of crocin on the H&E stained histopathological characteristics of joints and synovial tissues in CIA rats (×40). A–E: normal rats (A), control CIA rats (B), CIA rats treated with crocin at 10 (C), 20 (D), and 40 (E) mg/kg per day.
